# Exploiting nanoscale effects enables ultra-low temperature to produce porous silicon†

**DOI:** 10.1039/d1ra07212a

**Published:** 2021-11-01

**Authors:** Maximilian Yan, Siddharth V. Patwardhan

**Affiliations:** Department of Chemical and Biological Engineering, Green Nanomaterials Research Group, The University of Sheffield Mappin Street Sheffield S1 3JD UK s.patwardhan@sheffield.ac.uk

## Abstract

The magnesiothermic reduction (MgTR) of silica has been recently shown to produce porous silicon which can be used in applications such as photocatalysis and energy storage. MgTR typically requires ≥650 °C to achieve meaningful conversions. However, high temperatures are detrimental to the highly desired porosity of silicon, while also raising doubts over the sustainability of the process. In this work we show for the first time that the onset temperature of the MgTR is dependent on the particle size of the feedstock silica. Using both in-house synthesised and commercial silica, we have shown that only particles ≤20 nm are able to trigger the reaction at temperatures as low as 380 °C, well below a previously reported cut-off temperature of 500 °C, producing porous, crystalline silicon. The decrease in temperature requirement from ≥650 °C to 380 °C achieved with little modification to the overall process, without any additional downstream processing, presents significant implications for sustainable and economical manufacturing of porous silicon.

## Abstract 2

We show the first evidence of reduction of silica occurring at temperatures as low as 380 °C to produce porous silicon without sacrificing the porosity and yield, thus paving the way for sustainable manufacturing.

## Graphical abstract

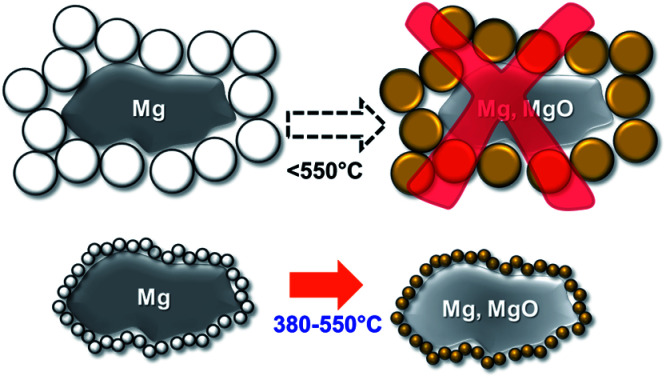

Porous silicon is a material which is heavily studied due to its structural and electronic properties, and lends itself well to applications such as photocatalytic water splitting,^1^ photoluminescence,^2^ and energy storage.^3^ However, for this material to become commercially accessible, a method of sustainably producing high quality silicon on a large scale is needed. It has recently been shown that the magnesiothermic reduction (MgTR) as a bulk method of producing porous silicon has great potential for scaling up, especially when compared to the carbothermal^4^ and electrochemical etching methods.^5^ The MgTR is a reaction in which powdered Mg, the reducing agent, is mixed with powdered silica and heated in a furnace under argon atmosphere. The reaction mixture is typically heated to 650 °C, and proceeds as shown in eqn (1):

1 2Mg_(s)_ + SiO_2(s)_ → 2MgO_(s)_ + Si_(s)_, Δ*H* = −291.62 kJ mol^−1^

The mixture, once removed from the furnace, is then immersed in HCl to remove any magnesium species. It was shown that higher reduction temperatures (≥650 °C) favour reaction completion,^6^ however higher temperatures are detrimental for the desired porosity due to sintering of nanocrystals. Below 650 °C, the yield begins to drop, until a cut-off temperature of 500 °C is reached, below which the reaction cannot proceed. Eutectic mixtures containing magnesium and aluminium have been shown to trigger the MgTR as low as 450 °C to produce a yield of silicon of 64 mol%.^7^ The drawback is that to form the eutectic, the metals have to be heated to 660 °C initially, which is the melting point of aluminium. Alternatively, a two-step reduction method has been demonstrated whereby the MgTR can progress at 300 °C, however this method requires the reactants to be heated initially to 650 °C to trigger the reaction.^8^

In addition to the need for carrying out MgTR at lower temperatures to maintain porosity, from scalability and sustainability perspectives, being able to lower the temperature requirement without sacrificing the porosity and most importantly, yield, would have positive implications to this process. Herein, for the first time, we demonstrate a method of achieving the MgTR reaction at low temperatures (<450 °C) without the use of eutectics or prior need of heating to 650 °C, to produce porous silicon with yields comparable to silicon made at temperatures above 650 °C. Through a systematic study, we demonstrate the importance and the effects of particle size of the silica feedstock on MgTR temperature.

It is well known that nanoparticles exhibit enhanced reactivity compared to their bulk counterparts due to an increase in surface energy from their high surface to volume ratio. It is thus possible to exploit this nanoscale effect by using silica nanoparticles in order to activate the MgTR reaction at low temperatures. However, the effects of silica particle sizes on MgTR have not been investigated yet. In this study, silica nanoparticles of sizes 500, 75 and 20 nm were synthesised using the Stöber method (see ESI†).^9^ These samples, referred to as S500, S75 and S20 respectively, were characterised using electron microscopy and N_2_ adsorption (Fig. 1 and Table S1†). As expected, the particles showed well-defined spherical morphology with a tight particle size distribution and they did not possess any internal porosity.

**Fig. 1:**
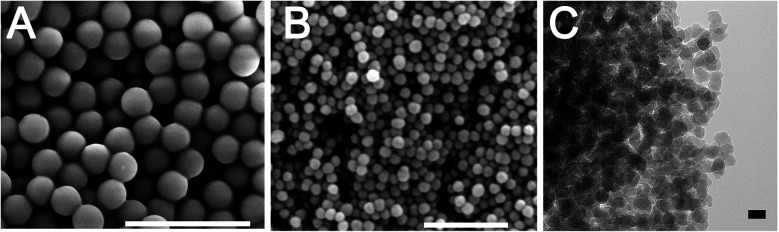
Electron microscopy images of (A) S500, (B) S75 and (C) S20. Scale bars of (A), (B) and (C) are 2 μm, 500 nm and 20 nm respectively.

The samples of silica were mixed with magnesium and reduced in a furnace at a range of temperatures between 450 and 750 °C. The reduction products were then washed with acid to remove Mg-containing species before characterising with SEM to study the morphology and XRD to detect the presence of crystalline silicon. When 500 nm silica (S500) were used, the absence of diffraction peaks for MgTR performed at 450 °C indicated that no crystalline product was formed at this temperature, consistent with the literature (Fig. 2A). MgTR temperature ≥550 °C was required to produce crystalline silicon as evident from peaks at 28, 47, 56, 68 and 76°, corresponding to 111, 220, 311, 400 and 331 crystal planes of Si, which matched the polycrystalline silicon standard. There were no peaks that correspond to MgO or unreacted Mg in any of the samples, thus confirming the effectiveness of the post-reaction acid washing.

**Fig. 2:**
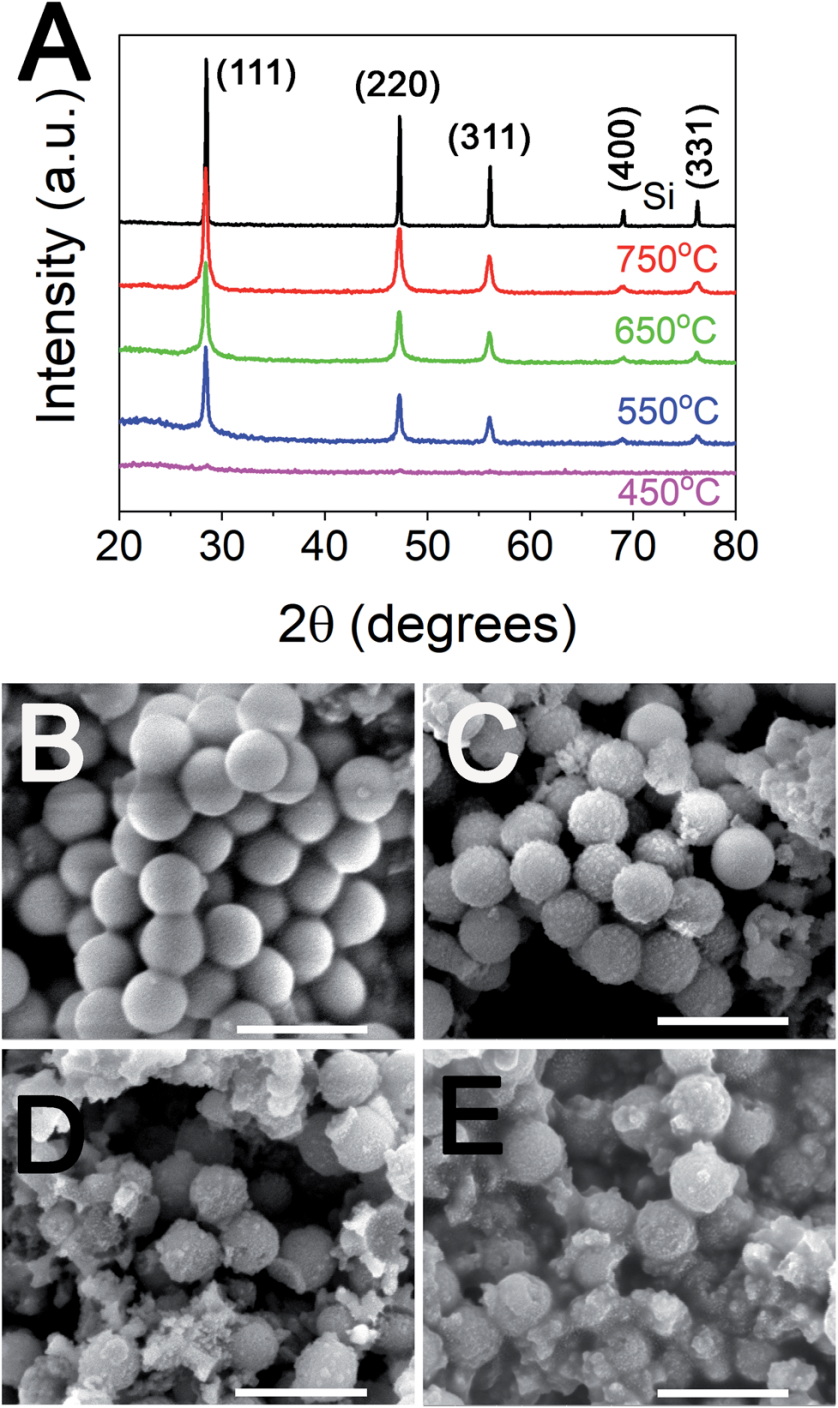
(A) Diffraction patterns of products obtained from reducing S500 at different temperatures, along with a polycrystalline silicon standard (Si, NIST Standard 640d). SEM images of 500 nm silica particles reduced at (B) 450 °C, (C) 550 °C, (D) 650 °C, and (E) 750 °C after washing in HCl are shown. Scale bars for SEM images are 1 μm.

The SEM images of S500 upon MgTR at 450 °C showed a lack of any change in the morphology, confirming that the particles have not undergone reduction. For temperatures of 550 °C, specks on the surface of the silica particles can be observed in the SEM images (Fig. 2B–D), which are consistent with the formation of silicon crystals reported in the literature.^10^ At 650 °C and 750 °C the spherical morphology was less obvious due to breakage of silica particles from extensive reduction and from the sintering of the newly formed crystalline silicon nanoparticles, again consistent with the literature.^10^ These silicon samples were porous in nature, *e.g.* S500 reduced at 650 °C was mesoporous with some microporosity and had a specific surface area of 179 m^2^ g^−1^ (see Fig. S2 and Table S2†). MgTR using 75 nm silica particles (S75) followed a similar trend, where crystalline silicon was produced at all reduction temperatures except 450 °C as shown in Fig. S1† (also see Fig. S3†).

In contrast to S500 and S75, when the 20 nm silica particles (S20) were used as the silica precursor for MgTR, crystalline silicon was produced at 450 °C as verified by characteristic sharp peaks in the XRD shown in Fig. 3A. The silicon produced had specific surface area of 184 m^2^ g^−1^, similar to the silicon obtained from reducing S500 at 650 °C. The formation of crystalline silica was further confirmed by TEM analysis shown in Fig. 3B. The lattice planes are shown by arrows in Fig. 3B to highlight the crystallites. Further analysis of the lattice planes observed in the TEM image was performed by calculating Fast Fourier Transform (FFT) to identify all possible *d*-spacings (see Fig. S4†). In total, four distinct *d*-spacings were found (tabulated in Table S3†) corresponding to (111), (220), (311) and (331) Miller indices. For brevity, only (111) is shown in Fig. 3B as an example. The strong corroboration between the XRD results and the TEM analysis further confirms the formation of crystalline silicon as the only crystalline phase. In order to determine whether the larger silica samples had produced amorphous Si, we quantified the yields by oxidising the MgTR products and any associated change in weight was measured using thermal gravimetric analysis (TGA). This method allows for quantification of both crystalline and amorphous silicon present in the bulk sample and hence it is preferred over surface analysis using techniques such as XPS.^10^

**Fig. 3:**
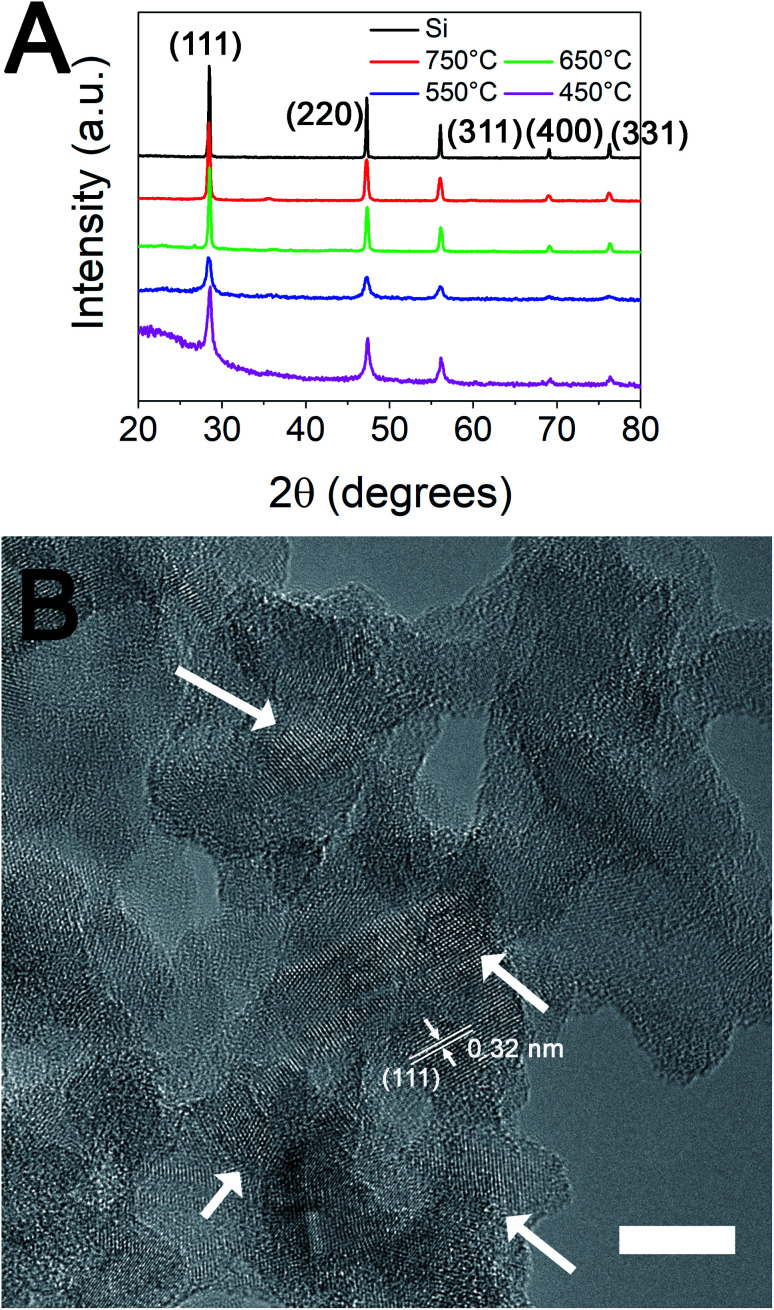
(A) Diffractograms of the reduction products of 20 nm silica particles. (B) TEM image of MgTR products obtained from S20 at 450 °C (scale bar is 10 nm). Arrows indicate the crystallites formed. Lattice fringes for (111) in a selected example is also shown.

When the MgTR was carried out at temperatures ≥550 °C, the yields of silicon obtained from different particle sizes were similar, irrespective of silica particle size (differences are within the measurement errors, see Fig. 4A). However, prominent differences were seen at 450 °C and below. 20 nm particles gave a yield of silicon of around 35 and 40 mol% for MgTR temperatures of 400 °C and 450 °C respectively, while at the same MgTR temperatures (<550 °C), S500 and S75 did not produced any detectable silicon. These results are in strong agreement with the results from XRD, where only 20 nm particles produced crystalline silicon at such low temperatures. Further lowering the MgTR temperature to 395 °C resulted in no detectable amount of silicon for any of the silica particles used (data not shown). This is the first evidence of MgTR of silica occurring at temperatures as low as 400 °C. These results also show for the first time that for MgTR reaction to occur at low temperatures, there is a particle size threshold: only the particles below this threshold will be able to initiate the MgTR reaction at sub-450 °C temperatures.

**Fig. 4:**
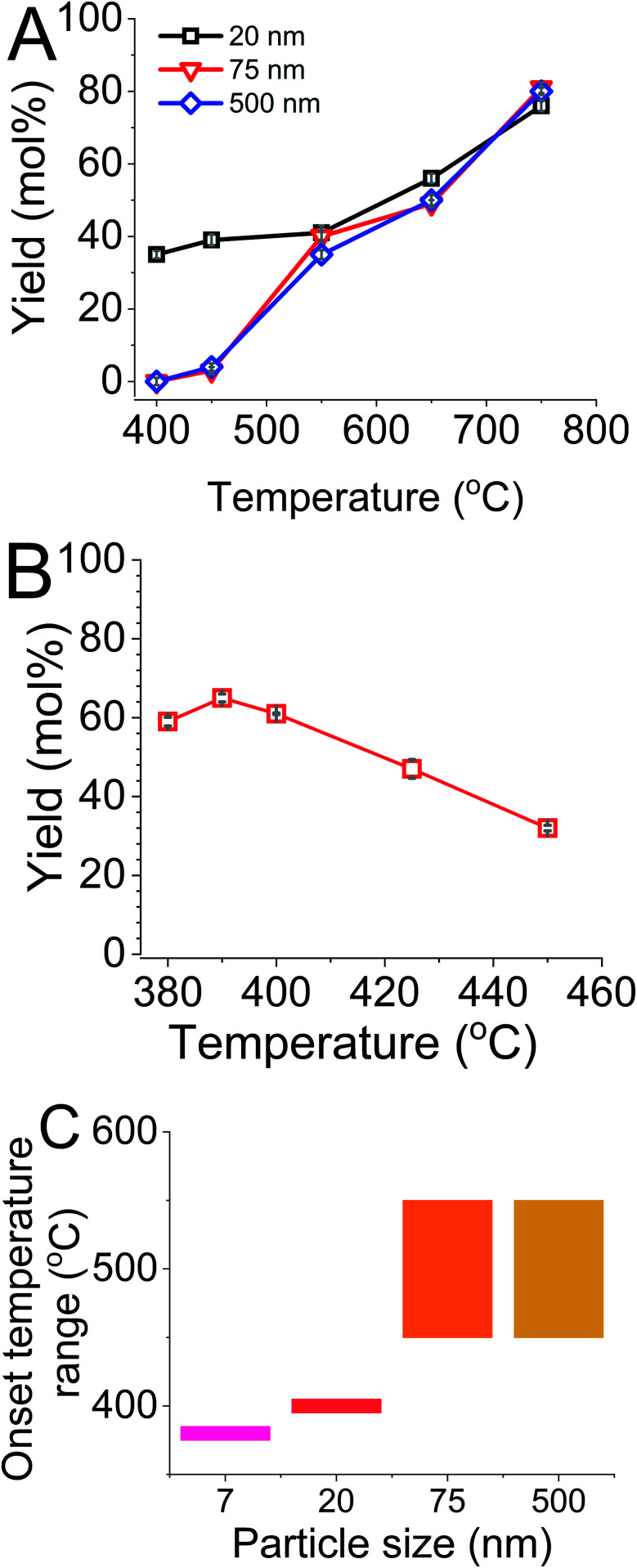
(A) The change in yield with MgTR temperature for different silica particle sizes. (B) Yields as a function of MgTR temperature for 7 nm silica. (C) Temperature brackets for the onset of MgTR with respect to the silica particle sizes.

This particle size threshold for lowering the activation temperature of MgTR is of particular interest. It is likely that using even smaller particles can lower the onset of MgTR further. In order to explore this possibility, 7 nm diameter fumed silica (purchased from Sigma, referred to as F7) was used to probe MgTR at low temperatures.^11^ F7 was able to produce silicon at 450 °C with a specific surface area of 177 m^2^ g^−1^ (again similar to that found for S500 at 650 °C). Interestingly, F7 produced silicon at temperatures as low as 380 °C (Fig. 4B) as confirmed with XRD (Fig. S5†). It is also worth noting that using this commercial silica further demonstrates that the activation of MgTR at lower temperatures exploiting the nanoscale effects is not limited to one type or source of silica particles. When MgTR was performed at 375 °C, silicon formation was not detected (data not shown). It was also observed that the yield of silicon from F7 at its onset temperature of 380 °C (∼60 mol%) was far higher than the yield recorded from S20 at its respective onset temperature of 400 °C (35 mol%). The yield of silicon from F7 declined from 390 °C as the temperature was raised to 450 °C (a decrease from 65 mol% to 32 mol%). It is likely that side reactions which consume the freshly formed silicon (*e.g.* the formation of magnesium silicide) are favoured at different temperatures.

These results show that the temperature needed to activate the MgTR reaction is dependent on the particle size of the feedstock silica. The smaller the particles, the lower the onset temperature (Fig. 4C). However, above a certain size (≥75 nm), the onset temperature did not reduce. We have therefore shown the first evidence of silica being converted to silicon *via* the MgTR reaction at a temperature as low as 380 °C, using only magnesium as the reducing agent (see Fig. 5). It is well known that nanoparticles exhibit enhanced reactivity compared to their bulk counterparts arising.^12,13^ This well-known nanoscale effect is likely to reduce the activation barrier for initiating MgTR reaction, and hence the energy input required to initiate the reaction is lower.^14^ This explains our findings where only the smaller silica nanoparticles (≤20 nm) react at temperatures ≤450 °C.

**Fig. 5:**
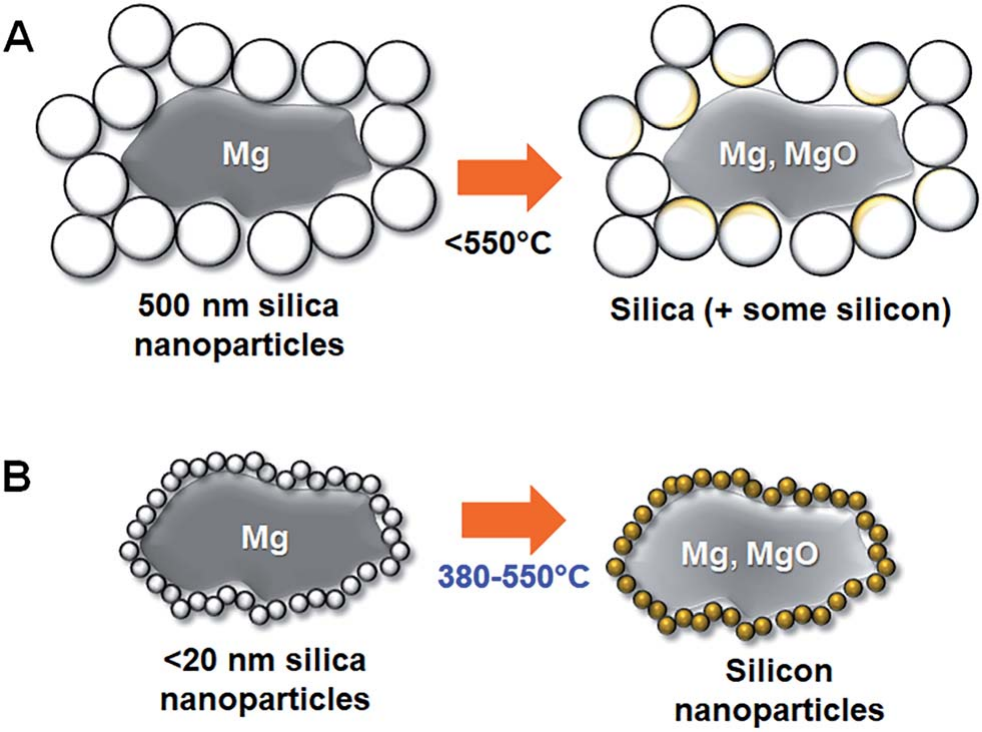
A schematic representation of the reduction of silica particles (shown in white with black edges) of sizes 500 nm (A) and <20 nm (B) in the presence of magnesium (shown in grey). Note that Mg particles used (10 s of μm) were significantly larger than silica particles. Only when small silica particles are used (B), reduction occurs at temperatures less than 550 °C, resulting in silicon and magnesia.

In conclusion, we have shown for the first time that the particle size of the feedstock silica affects the temperature requirement of MgTR reaction due to the nanoscale phenomenon. The MgTR has an onset temperature which is dependent on particle size. Stöber particles ≥75 nm have a reduction onset temperature of ≥550 °C. For particles ≤20 nm, the onset temperature decreases with particle size, where 20 nm particles were able to reduce at 400 °C while 7 nm particles are able to reduce at 380 °C. This study shows that it is possible to produce silicon at lower temperatures with minimal compromise to yield. Further studies are required to understand and manipulate the mechanisms of this low temperature process. The findings in this paper paves the way for the development of a sustainable and scalable MgTR reaction strategy to produce porous silicon for photocatalysis, energy storage and other applications.

## Experimental methods

### Materials and reagents

Magnesium powder (99.98%) of −325 mesh grade and ethanol (absolute) were purchased from VWR. Ammonium hydroxide solution (28%), tetraethyl orthosilicate (TEOS 99.9%) and hydrochloric acid (HCl) were purchased from Sigma-Aldrich. Fumed silica was also purchased and used as received from Sigma-Aldrich.

### Stöber silica synthesis

Silica nanospheres of various sizes were made *via* the Stöber method.^9^ Ethanol (Absolute, VWR) and TEOS (Sigma-Aldrich) were added to volumetric flask and mixed with a Teflon coated magnetic stirring bar. After 10 minutes of mixing, deionised water was added, and following 10 more minutes of mixing, 28% ammonium hydroxide solution was added dropwise to the solution. The amounts of ethanol and ammonium hydroxide were varied to achieve the desired concentration of ammonium hydroxide. The solution was stirred for a further 24 hours at 20 °C, followed by centrifugation at 5000 rpm for 15 minutes to separate the particles from the solution. The particles were rinsed with deionised water to remove excess ethanol, ammonium hydroxide and TEOS, then centrifuged once more at 5000 rpm for 15 minutes, before being dried overnight at 80 °C. Smaller particles (≤20 nm) were separated using a dialysis membrane with a 14 kDa molecular weight cut-off, immersed in a deionised water bath. The conductivity (inverse resistance) of the water bath was measured at 4-hour intervals using a multimeter on 2 MΩ resistance setting, and when two consecutive readings were the same, the water in the bath was removed and replenished with fresh de-ionised water to introduce a new diffusion gradient. This was repeated several times until the conductivity was unchanging, then the particles within the dialysis membrane were separated by drying on an evaporating dish.

### Silicon synthesis

The preparation of the precursors for MgTR was according to our previously reported method^6^ using magnesium powder (325 mesh, 99.8%, Alfa Aesar) and the synthesised Stöber silica nanospheres a weight ratio of 1 : 1, giving the stoichiometric mole ratio of 2.5 : 1. In every reduction, 0.5 g of magnesium and 0.5 g of silica were used. In mixed size silica precursors, the total mass of silica used in each reaction remained the same. A ramp rate of 1 °C min^−1^ was used for all reductions. Samples were heated at maximum temperatures for a duration of 6 hours then cooled naturally to room temperature.

### Analytical techniques

*Scanning electron microscopy (SEM)* images of the as synthesised and reduced Stöber particles were taken using FEI Inspect F electron microscope. Samples were prepared by dispersing dry silica powder in ethanol and drop casted onto carbon coated aluminium sheets, then gold coated using a sputter coater. *Transmission electron microscopy (TEM) imaging* was performed on a Jeol R005 80–300 kV transmission electron microscope. Samples were dispersed in ethanol by ultrasonication then drop-casted onto 400 mesh copper TEM grids coated with lacey carbon films (Agar Scientific). TEM images were analysed using ImageJ software to identify crystal cell parameters by calculating FFT to provide *d*-spacings. *Powder X-ray diffraction (XRD)* technique was performed using the Stoe Stadi P (CuIP) diffractometer, with copper K_α1_ radiation of wavelength 1.5406 Å. 5 diffractograms were collected for each sample within the 2*θ* range of 1 and 120°, each taking 6 minutes. The diffractograms were combined to reduce background noise, then analysed using WinXPow software. *Thermal gravimetric analysis* was performed using a PerkinElmer TGA 4000, under continuous flow of oxygen. To ensure complete oxidation of the silicon present, samples were heated from 20 °C to 950 °C at a rate of 40 °C min^−1^, holding at maximum temperature for 24 hours, before cooling at 40 °C min^−1^ to room temperature. *Surface area and pore size measurements* were performed using a Micromeritics Tristar 3000. Samples were degassed under vacuum at 120 °C for 24 hours, then their weights were recorded. Warm and cold free space measurements were taken using He before and after immersing in liquid nitrogen, respectively. Adsorption and desorption isotherms were then collected within the range of 0.001 and 0.999 *P*/*P*_0_ using N_2_ as the adsorbent whilst samples were immersed in a liquid nitrogen-filled dewar. Specific surface area was deduced from the isotherms by fitting with the BET model. Pore volumes and average pore diameters were deduced using the BJH model on the desorption branch.

## Conflicts of interest

There are no conflicts to declare.

## Supplementary Material

RA-011-D1RA07212A-s001
