# Supplementary material for: Exploiting nanoscale effects enables ultra-low temperature to produce porous silicon
Source: RSC Adv. 2021 Nov 1;11(56):35182–6. doi: 10.1039/d1ra07212a (PMC9043007; doi:10.1039/d1ra07212a)
Supplement: RA-011-D1RA07212A-s001 [file RA-011-D1RA07212A-s001.pdf]

Electronic Supplementary Material (ESI) for RSC Advances.
This journal is © The Royal Society of Chemistry 2021

Electronic Supplementary Information

Exploiting nanoscale effects enables ultra-low temperature to produce porous silicon

*Maximilian Yan and Siddharth V. Patwardhan**

Department of Chemical and Biological Engineering, Green Nanomaterials Research Group, The University of Sheffield, Mappin Street, Sheffield S1 3JD, UK.
*s.patwardhan@sheffield.ac.uk

Table S1: Concentration of NH_3 used in Stöber silica synthesis and the resulting silica particle sizes measured by TEM.

$[NH_3]$ (M)	Particle diameter (nm)	Internal specific surface area (m^2/g)
0.01	20 ± 2	45
0.05	75 ± 8	9
1.13	500 ± 17	6

Table S2: Properties of silica and their reduction products at different temperatures are summarised.

**Sample**	**Silicon Yield (mol%)**	**Specific surface area (m^2/g)[a]**	**Total pore volume (cm^3/g)**
S20	n/a *	189	0.79
S500	n/a *	7	0.01
S20 450°C	35	184	0.24
S20 650°C	56	92	0.19
S500 650°C	50	179	0.29
F7	n/a *	424	0.7
F7 380°C	58	70	0.16
F7 450°C	31	177	0.47

[a] This is total surface area (internal and external).
* This is the feedstock silica samples which has not undergone reduction and hence a yield of silicon is not applicable.

Table S3: Crystal planes identified in Figure S4 and associated d-spacings, 2θ angles and miller indices.

**d-spacing (nm)**	**2θ (degrees)**	**Miller Indices**
0.32	28	(111)
0.20	48	(220)
0.17	56	(311)
0.14	76	(331)

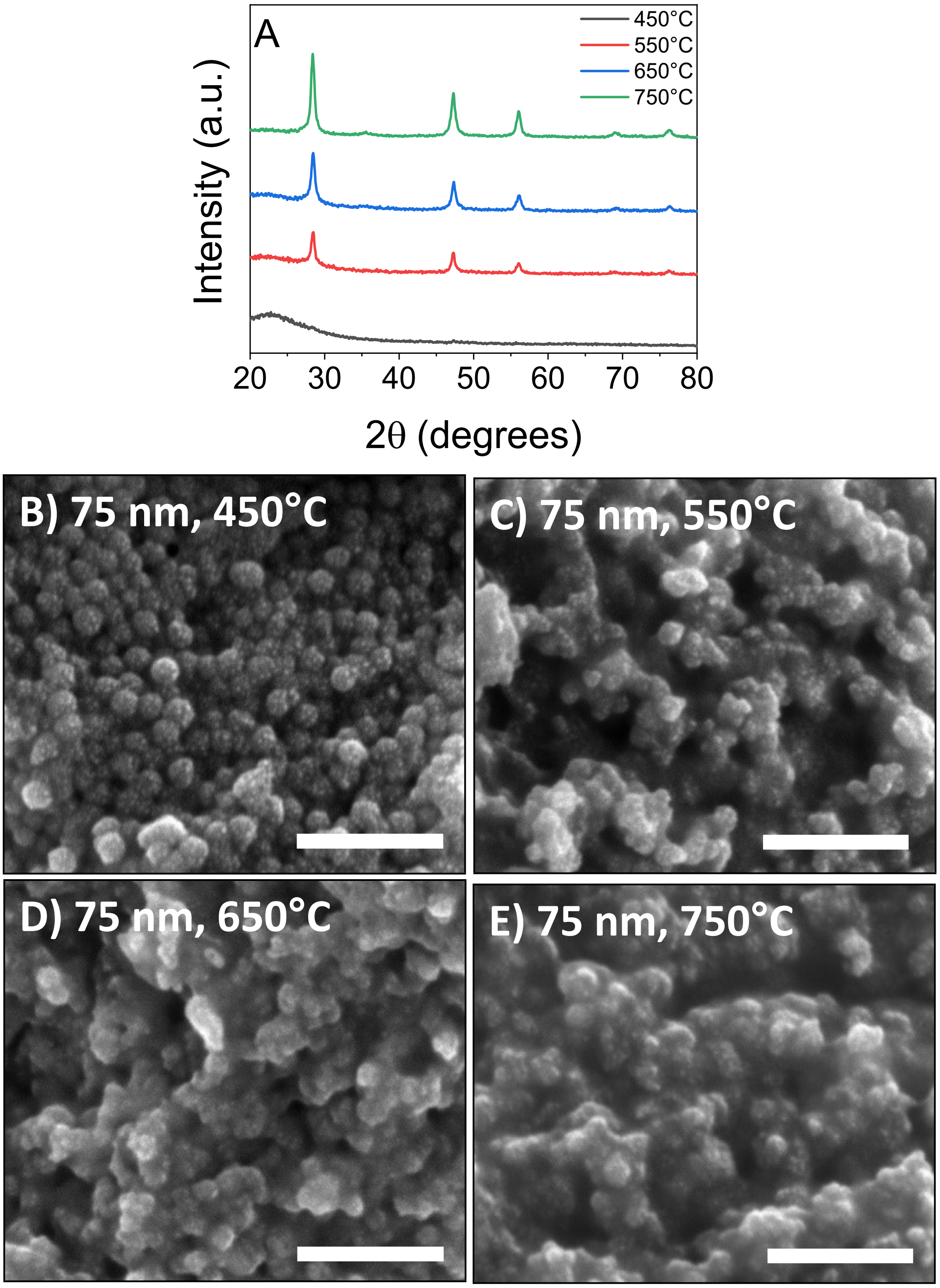

Figure S1: (A) XRD and (B-E) SEM images of 75 nm particles (S75) reduced at different temperatures. Scale bars of SEM images are 500 nm. All data was collected on samples after HCl etching.

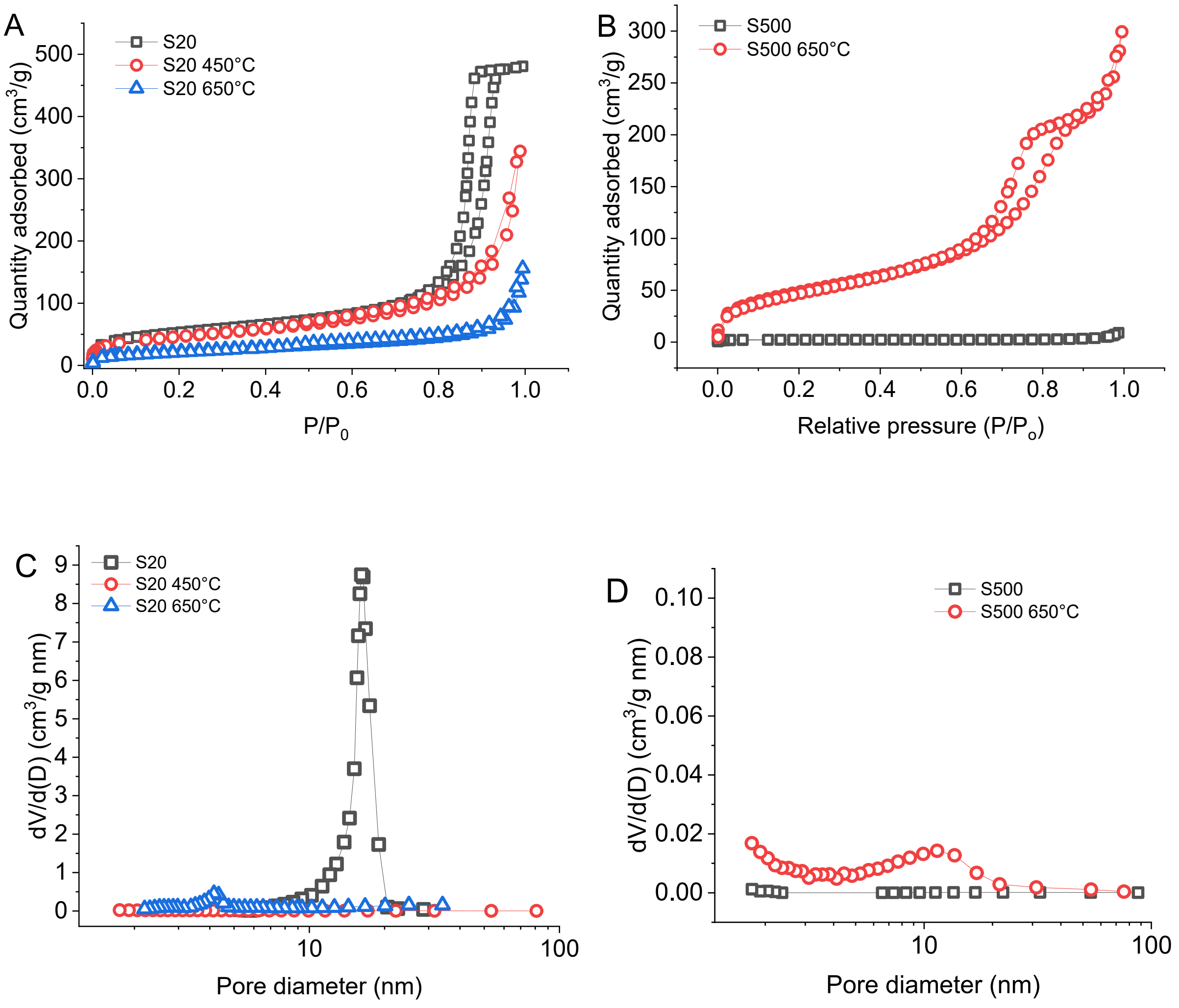

Figure S2: The isotherms for pristine and reduced A) S20 particles* and B) S500 particles are given. C) The BJH desorption pore sizes are shown for C) S20 and D) S500 samples, both reduced and unreduced samples. *While S20 does not have any internal surface area, the data in parts (A) and (C) include external surface area.

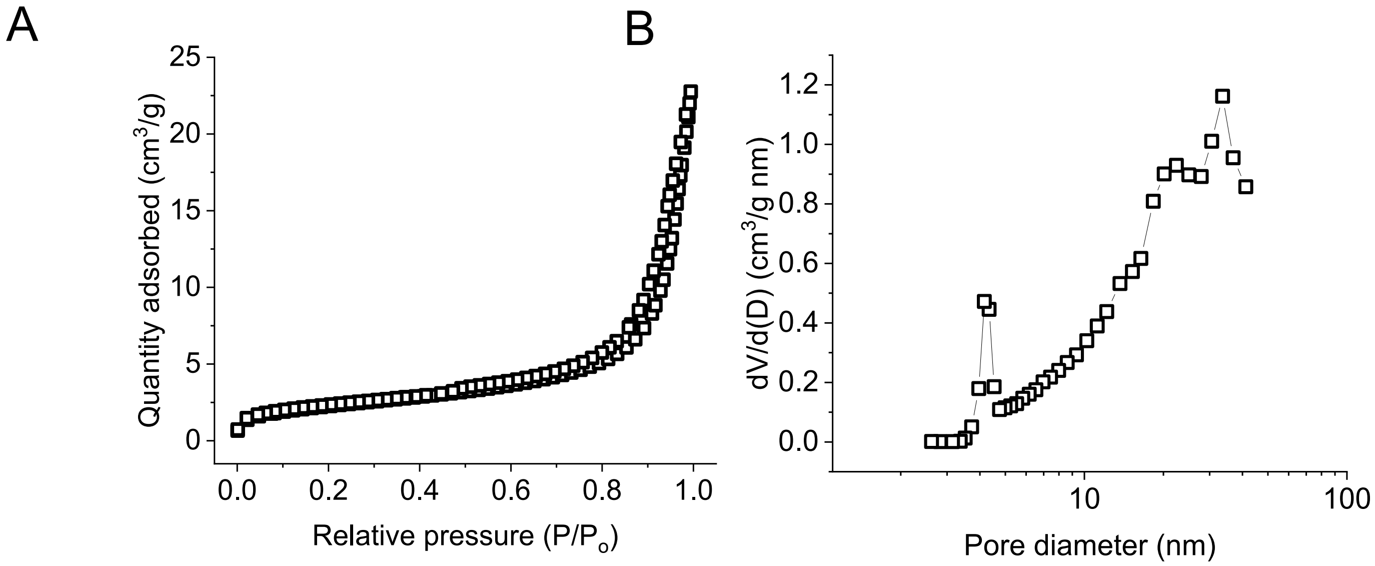

Figure S3: A) Gas adsorption isotherm and B) pore size distribution of 75 nm particles (S75) reduced at 650°C.

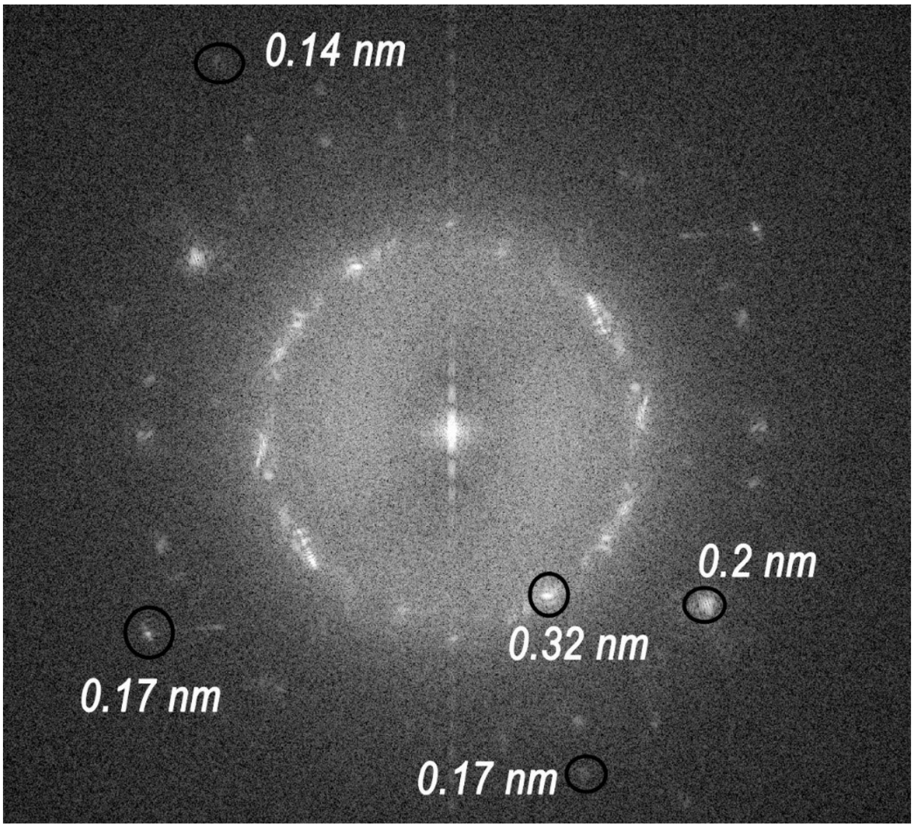

Fig. S4: FFT analysis of the TEM image shown in Figure 3B. d-spacings for all identified crystal planes are shown on the image and associated parameters are listed in Table S3.

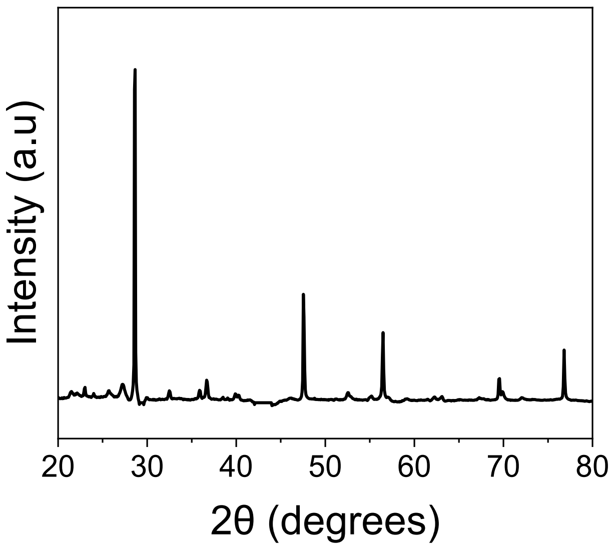

Fig. S5: XRD of the reduction of F7 at 380°C after washing in HCl.
